# Supplementary material for: Heterotypic Stressors Unmask Behavioral Influences of PMAT Deficiency in Mice
Source: Int J Mol Sci. 2023 Nov 18;24(22):16494. doi: 10.3390/ijms242216494 (PMC10671398; doi:10.3390/ijms242216494)
Supplement: Supplementary file 1 [file ijms-24-16494-s001.zip › ijms-2627478-supplementary.pdf]

Supplemental Material for:

Heterotypic stressors unmask behavioral influences of PMAT deficiency in mice

Brady L Weber, Marissa M Nicodemus, Allianna K Hite, Isabella R Spalding, Jasmin N Beaver, Lauren R Scrimshaw, Sarah K Kassis, Julie M Reichert, Matthew T Ford, Cameron N Russell, Elayna M Hallal, T Lee Gilman*

Department of Psychological Sciences
Brain Health Research Institute
Healthy Communities Research Institute
Kent State University, Kent, OH, USA

*Corresponding author
lgilman1@kent.edu
600 Hilltop Dr.
209 Kent Hall
Kent State University
Kent, OH, USA 44242

Supplemental Table S1. Repeated measures ANOVAs of Phase 1 Cued Female data.

Phase 1 Cued Females

Training

	F statistic	*p*	*partial η²*
Genotype	F(1,35)=0.531	0.471	0.015
Swim	F(1,35)=0.003	0.957	0.000
Genotype × Swim	F(1,35)=0.881	0.354	0.025
**Time**	**F(3.122,109.266)=183.4**	**<0.001**	**0.840**
Time × Genotype	F(3.122,109.266)=0.676	0.574	0.019
Time × Swim	F(3.122,109.266)=0.585	0.633	0.016
Time × Genotype × Swim	F(3.122,109.266)=0.964	0.415	0.027

Expression Testing & Extinction Training

	F statistic	*p*	*partial η²*
Genotype	F(1,35)=0.471	0.497	0.013
Swim	F(1,35)=0.455	0.504	0.013
Genotype × Swim	F(1,35)=0.751	0.392	0.021
Time	F(9.728,340.487)=8.714	<0.001	0.199
**Time × Genotype**	**F(9.728,340.487)=3.865**	**<0.001**	**0.099**
Time × Swim	F(9.728,340.487)=0.802	0.624	0.022
Time × Genotype × Swim	F(9.728,340.487)=1.138	0.334	0.031

Extinction Retention Testing & More Extinction Training

	F statistic	*p*	*partial η²*
Genotype	F(1,35)=1.000	0.324	0.028
Swim	F(1,35)=0.477	0.494	0.013
Genotype × Swim	F(1,35)=0.508	0.481	0.014
**Time**	**F(9.774,342.097)=8.024**	**<0.001**	**0.186**
Time × Genotype	F(9.774,342.097)=1.034	0.414	0.029
Time × Swim	F(9.774,342.097)=1.523	0.131	0.042
Time × Genotype × Swim	F(9.774,342.097)=1.191	0.296	0.033

Context Fear Expression - Timecourse

	F statistic	*p*	*partial η²*
Genotype	F(1,35)=0.001	0.976	0.000
Swim	F(1,35)=0.110	0.742	0.003
Genotype × Swim	F(1,35)=0.027	0.871	0.001
**Time**	**F(6.926,242.403)=5.297**	**<0.001**	**0.131**
Time × Genotype	F(6.926,242.403)=0.641	0.720	0.018
Time × Swim	F(6.926,242.403)=0.586	0.765	0.016

Time × Genotype × Swim	F(6.926,242.403)=0.531	0.809	0.015

Cued Fear Renewal

	F statistic	***p***	***partial η²***
Genotype	F(1,35)=0.009	0.926	0.000
Swim	F(1,35)=0.075	0.786	0.002
Genotype × Swim	F(1,35)=0.421	0.521	0.012
**Time**	**F(3.712,129.923)=12.40**	**<0.001**	**0.262**
Time × Genotype	F(3.712,129.923)=0.574	0.670	0.016
Time × Swim	F(3.712,129.923)=0.745	0.554	0.021
Time × Genotype × Swim	F(3.712,129.923)=0.184	0.937	0.005

Supplemental Table S2. Repeated measures ANOVAs of Phase 1 Cued Male data.

Phase 1 Cued Males

Training

	F statistic	*p*	*partial η²*
Genotype	F(1,54)=3.148	0.082	0.055
Swim	F(1,54)=1.825	0.182	0.033
Genotype × Swim	F(1,54)=0.166	0.685	0.003
**Time**	**F(3.156,170.433)=205.7**	**<0.001**	**0.792**
Time × Genotype	F(3.156,170.433)=1.554	0.200	0.028
Time × Swim	F(3.156,170.433)=0.475	0.710	0.009
Time × Genotype × Swim	F(3.156,170.433)=0.477	0.708	0.009

Expression Testing & Extinction Training

	F statistic	*p*	*partial η²*
Genotype	F(1,54)=1.051	0.310	0.019
Swim	F(1,54)=0.074	0.787	0.001
Genotype × Swim	F(1,54)=2.257	0.139	0.040
**Time**	**F(10.594,572.057)=9.786**	**<0.001**	**0.153**
Time × Genotype	F(10.594,572.057)=0.538	0.872	0.010
Time × Swim	F(10.594,572.057)=0.554	0.860	0.010
Time × Genotype × Swim	F(10.594,572.057)=0.718	0.716	0.013

Extinction Retention Testing & More Extinction Training

	F statistic	*p*	*partial η²*
Genotype	F(1,53)=0.173	0.679	0.003
Swim	F(1,53)=0.035	0.852	0.001
Genotype × Swim	F(1,53)=1.543	0.220	0.028
**Time**	**F(10.125,536.64)=11.09**	**<0.001**	**0.173**
Time × Genotype	F(10.125,536.64)=1.004	0.439	0.019
Time × Swim	F(10.125,536.64)=1.339	0.205	0.025
Time × Genotype × Swim	F(10.125,536.64)=0.633	0.788	0.012

Context Fear Expression

	F statistic	*p*	*partial η²*
Genotype	F(1,49)=0.250	0.619	0.005
Swim	F(1,49)=0.684	0.412	0.014
Genotype × Swim	F(1,49)=1.147	0.289	0.023
**Time**	**F(10.889,533.558)=11.58**	**<0.001**	**0.191**
Time × Genotype	F(10.889,533.558)=0.631	0.801	0.013
Time × Swim	F(10.889,533.558)=1.197	0.286	0.024

Time × Genotype × Swim	F(10.889,533.558)=1.319	0.210	0.026

Cued Fear Renewal

	F statistic	***p***	***partial η²***
Genotype	F(1,49)=0.033	0.856	0.001
Swim	F(1,49)=0.874	0.354	0.018
Genotype × Swim	F(1,49)=0.081	0.777	0.002
**Time**	**F(3.82,187.198)=11.17**	**<0.001**	**0.186**
Time × Genotype	F(3.82,187.198)=0.190	0.938	0.004
Time × Swim	F(3.82,187.198)=0.182	0.942	0.004
Time × Genotype × Swim	F(3.82,187.198)=0.999	0.407	0.020

Supplemental Table S3. Repeated measures ANOVAs of Phase 1 Context Female data.

Phase 1 Context Females

Training

	F statistic	***p***	***partial η²***
Genotype	F(1,34)=0.904	0.348	0.026
Swim	F(1,34)=0.014	0.907	0.000
Genotype × Swim	F(1,34)=0.061	0.806	0.002
**Time**	**F(3.626,123.285)=125.3**	**<0.001**	**0.787**
Time × Genotype	F(3.626,123.285)=0.252	0.893	0.007
Time × Swim	F(3.626,123.285)=0.818	0.506	0.023
Time × Genotype × Swim	F(3.626,123.285)=0.412	0.782	0.012

Context Fear Expression

	F statistic	***p***	***partial η²***
Genotype	*F(1,34)=2.936*	*0.096*	*0.079*
Swim	F(1,34)=0.540	0.468	0.016
Genotype × Swim	F(1,34)=0.555	0.462	0.016
**Time**	**F(9.138,310.686)=8.874**	**<0.001**	**0.207**
Time × Genotype	F(9.138,310.686)=1.369	0.200	0.039
Time × Swim	F(9.138,310.686)=0.494	0.881	0.014
Time × Genotype × Swim	F(9.138,310.686)=0.984	0.454	0.028

Supplemental Table S4. Repeated measures ANOVAs of Phase 1 Context Male data.

Phase 1 Context Males

Training

	F statistic	*p*	*partial η²*
Genotype	F(1,39)=0.553	0.462	0.014
Swim	F(1,39)=2.221	0.144	0.054
Genotype × Swim	F(1,39)=0.081	0.777	0.002
**Time**	**F(3.226,125.803)=12.92**	**<0.001**	**0.249**
Time × Genotype	F(3.226,125.803)=1.424	0.237	0.035
Time × Swim	F(3.226,125.803)=0.441	0.738	0.011
Time × Genotype × Swim	F(3.226,125.803)=0.158	0.934	0.004

Context Fear Expression

	F statistic	*p*	*partial η²*
**Genotype**	**F(1,39)=4.555**	**0.039**	**0.105**
Swim	F(1,39)=0.027	0.871	0.001
Genotype × Swim	F(1,39)=1.280	0.265	0.032
**Time**	**F(9.546,372.282)=13.94**	**<0.001**	**0.263**
Time × Genotype	F(9.546,372.282)=1.142	0.331	0.028
Time × Swim	F(9.546,372.282)=1.319	0.221	0.033
Time × Genotype × Swim	F(9.546,372.282)=0.840	0.586	0.021

Supplemental Table S5. Repeated measures ANOVAs of Phase 2 Cued Female data.

Phase 2 Cued Females

Training

	F statistic	*p*	*partial η^2*
Genotype	F(1,41)=0.676	0.416	0.016
Swim	F(1,41)=1.273	0.266	0.030
Genotype × Swim	F(1,41)=0.922	0.343	0.022
**Time**	**F(3.605,147.811)=159.1**	**<0.001**	**0.795**
Time × Genotype	F(3.605,147.811)=0.679	0.593	0.016
Time × Swim	F(3.605,147.811)=0.970	0.420	0.023
Time × Genotype × Swim	F(3.605,147.811)=0.169	0.942	0.004

Expression Testing & Extinction Training

	F statistic	*p*	*partial η^2*
Genotype	F(1,41)=0.299	0.587	0.007
Swim	F(1,41)=0.036	0.850	0.001
Genotype × Swim	F(1,41)=0.951	0.335	0.023
**Time**	**F(8.741,358.393)=6.623**	**<0.001**	**0.139**
Time × Genotype	F(8.741,358.393)=0.852	0.566	0.020
Time × Swim	F(8.741,358.393)=0.726	0.681	0.017
Time × Genotype × Swim	F(8.741,358.393)=0.437	0.911	0.011

Extinction Retention Testing & More Extinction Training

	F statistic	*p*	*partial η^2*
Genotype	F(1,35)=0.400	0.531	0.011
Swim	F(1,35)=0.093	0.763	0.003
Genotype × Swim	F(1,35)=0.009	0.924	0.000
**Time**	**F(8.864,310.252)=11.15**	**<0.001**	**0.242**
Time × Genotype	F(8.864,310.252)=0.590	0.803	0.017
Time × Swim	F(8.864,310.252)=0.822	0.594	0.023
Time × Genotype × Swim	F(8.864,310.252)=1.133	0.339	0.031

Context Fear Expression

	F statistic	*p*	*partial η^2*
Genotype	F(1,41)=0.256	0.615	0.006
Swim	F(1,41)=0.498	0.484	0.012
Genotype × Swim	F(1,41)=0.060	0.807	0.001
**Time**	**F(10.762,441.257)=5.923**	**<0.001**	**0.126**
Time × Genotype	F(10.762,441.257)=1.001	0.444	0.024
Time × Swim	F(10.762,441.257)=1.15	0.321	0.027

Time × Genotype × Swim	F(10.762,441.257)=1.064	0.389	0.025

Cued Fear Renewal

	F statistic	***p***	***partial η²***
Genotype	F(1,39)=0.637	0.430	0.016
Swim	F(1,39)=0.050	0.825	0.001
Genotype × Swim	F(1,39)=0.066	0.799	0.002
Time	F(3.57,139.219)=11.74	<0.001	0.231
Time × Genotype	F(3.57,139.219)=0.690	0.584	0.017
**Time × Swim**	**F(3.57,139.219)=3.592**	**0.011**	**0.084**
Time × Genotype × Swim	F(3.57,139.219)=0.282	0.870	0.007

Supplemental Table S6. Repeated measures ANOVAs of Phase 2 Cued Male data.

Phase 2 Cued Males

Training

	F statistic	*p*	*partial η^2*
Genotype	F(1,36)=0.033	0.858	0.001
Swim	F(1,36)=0.140	0.710	0.004
Genotype × Swim	F(1,36)=1.226	0.276	0.033
**Time**	**F(2.700,97.197)=123.1**	**<0.001**	**0.774**
Time × Genotype	F(2.700,97.197)=1.581	0.203	0.042
Time × Swim	F(2.700,97.197)=0.872	0.449	0.024
Time × Genotype × Swim	F(2.700,97.197)=0.978	0.400	0.026

Expression Testing & Extinction Training

	F statistic	*p*	*partial η^2*
Genotype	F(1,30)=0.196	0.661	0.006
Swim	F(1,30)=1.649	0.209	0.052
Genotype × Swim	F(1,30)=0.725	0.401	0.024
**Time**	**F(8.933,267.996)=10.55**	**<0.001**	**0.260**
Time × Genotype	F(8.933,267.996)=1.029	0.417	0.033
Time × Swim	F(8.933,267.996)=0.987	0.451	0.032
Time × Genotype × Swim	F(8.933,267.996)=1.631	0.107	0.052

Extinction Retention Testing & More Extinction Training

	F statistic	*p*	*partial η^2*
**Genotype**	**F(1,38)=6.914**	**0.012**	**0.154**
Swim	F(1,38)=0.052	0.820	0.001
Genotype × Swim	F(1,38)=1.033	0.316	0.026
**Time**	**F(7.79,296.005)=8.583**	**<0.001**	**0.184**
Time × Genotype	F(7.79,296.005)=0.955	0.470	0.025
Time × Swim	F(7.79,296.005)=1.211	0.293	0.031
Time × Genotype × Swim	F(7.79,296.005)=1.249	0.272	0.032

Context Fear Expression

	F statistic	*p*	*partial η^2*
**Genotype**	**F(1,37)=4.175**	**0.048**	**0.101**
Swim	F(1,37)=0.273	0.604	0.007
Genotype × Swim	F(1,37)=2.728	0.107	0.069
**Time**	**F(9.633,356.405)=5.218**	**<0.001**	**0.124**
Time × Genotype	F(9.633,356.405)=0.390	0.947	0.010
Time × Swim	F(9.633,356.405)=1.470	0.152	0.038

Time × Genotype × Swim	F(9.633,356.405)=1.183	0.302	0.031

Cued Fear Renewal

	F statistic	***p***	***partial η²***
Genotype	F(1,34)=0.164	0.688	0.005
Swim	F(1,34)=1.950	0.172	0.054
Genotype × Swim	F(1,34)=0.625	0.435	0.018
**Time**	**F(3.835,130.383)=24.30**	**<0.001**	**0.417**
Time × Genotype	F(3.835,130.383)=0.851	0.492	0.024
Time × Swim	F(3.835,130.383)=0.898	0.464	0.026
Time × Genotype × Swim	F(3.835,130.383)=1.335	0.262	0.038

Supplemental Table S7. Repeated measures ANOVAs of Phase 2 Context Female data.

Phase 2 Context Females

Training

	F statistic	*p*	*partial η^2*
Genotype	F(1,34)=0.713	0.404	0.021
Swim	F(1,34)=0.346	0.560	0.010
Genotype × Swim	F(1,34)=0.065	0.800	0.002
**Time**	**F(3.029,102.984)=76.52**	**<0.001**	**0.692**
Time × Genotype	F(3.029,102.984)=1.116	0.346	0.032
Time × Swim	F(3.029,102.984)=1.963	0.124	0.055
Time × Genotype × Swim	F(3.029,102.984)=0.978	0.407	0.028

Context Fear Expression

	F statistic	*p*	*partial η^2*
Genotype	F(1,30)=0.362	0.552	0.012
Swim	F(1,30)=2.066	0.161	0.064
Genotype × Swim	F(1,30)=1.549	0.223	0.049
**Time**	**F(9.209,276.265)=8.773**	**<0.001**	**0.226**
Time × Genotype	F(9.209,276.265)=1.094	0.367	0.035
Time × Swim	F(9.209,276.265)=0.985	0.454	0.032
Time × Genotype × Swim	F(9.209,276.265)=1.251	0.263	0.040

Supplemental Table S8. Repeated measures ANOVAs of Phase 2 Context Male data.

Phase 2 Context Males

Training

	F statistic	***p***	***partial η²***
Genotype	F(1,33)=0.042	0.839	0.001
Swim	F(1,33)=0.075	0.786	0.002
Genotype × Swim	F(1,33)=0.180	0.674	0.005
**Time**	**F(3.284,108.38)=66.40**	**<0.001**	**0.668**
Time × Genotype	F(3.284,108.38)=0.190	0.917	0.006
Time × Swim	F(3.284,108.38)=0.979	0.411	0.029
Time × Genotype × Swim	F(3.284,108.38)=1.062	0.372	0.031

Context Fear Expression

	F statistic	***p***	***partial η²***
Genotype	F(1,33)=0.524	0.474	0.016
Swim	F(1,33)=0.024	0.877	0.001
Genotype × Swim	*F(1,33)=3.400*	*0.074*	*0.093*
**Time**	**F(8.625,284.634)=14.87**	**<0.001**	**0.311**
Time × Genotype	F(8.625,284.634)=1.447	0.171	0.042
Time × Swim	F(8.625,284.634)=1.160	0.322	0.034
Time × Genotype × Swim	F(8.625,284.634)=0.763	0.646	0.023

Supplemental Table S9. Three-way ANOVAs of log-transformed corticosterone levels taken 2 h following final testing (Phase 1 – swim stress; Phase 2 – context testing & cued fear renewal).

**Phase 1 Cued**			
	F statistic	***p***	***partial η²***
Genotype	F(1,82)=0.962	0.330	0.012
**Sex**	**F(1,82)=17.77**	**<0.001**	**0.178**
Swim	F(1,82)=0.301	0.584	0.004
Genotype × Sex	F(1,82)=0.119	0.731	0.001
Genotype × Swim	F(1,82)=0.170	0.681	0.002
Sex × Swim	F(1,82)=0.000	0.992	0.000
Genotype × Sex × Swim	F(1,82)=0.639	0.426	0.008

**Phase 1 Context**			
	F statistic	***p***	***partial η²***
Genotype	F(1,72)=2.007	0.161	0.027
Sex	F(1,72)=45.11	<0.001	0.385
Swim	F(1,72)=8.382	0.005	0.104
Genotype × Sex	F(1,72)=4.839	0.031	0.063
Genotype × Swim	F(1,72)=0.239	0.626	0.003
Sex × Swim	F(1,72)=5.938	0.017	0.076
**Genotype × Sex × Swim**	**F(1,72)=6.029**	**0.016**	**0.077**

**Phase 2 Cued**			
	F statistic	***p***	***partial η²***
Genotype	F(1,79)=2.285	0.135	0.028
**Sex**	**F(1,79)=17.40**	**<0.001**	**0.180**
Swim	F(1,79)=0.967	0.328	0.012
Genotype × Sex	F(1,79)=1.157	0.285	0.014
Genotype × Swim	F(1,79)=1.748	0.190	0.022
Sex × Swim	F(1,79)=0.390	0.534	0.005
Genotype × Sex × Swim	F(1,79)=1.235	0.270	0.015

**Phase 2 Context**			
	F statistic	***p***	***partial η²***
Genotype	F(1,63)=0.001	0.979	0.000
Sex	F(1,63)=1.437	0.235	0.022
Swim	F(1,63)=0.670	0.416	0.011
Genotype × Sex	F(1,63)=0.186	0.668	0.003
**Genotype × Swim**	**F(1,63)=4.377**	**0.040**	**0.065**
Sex × Swim	F(1,63)=0.100	0.753	0.002

Genotype × Sex × Swim	$F(1,63)=0.282$	0.597	0.004

Supplemental Table S10. Two-way ANOVAs for behaviors during swim stress in Phase 1 Cued mice.

Phase 1 Cued

Swimming

	F statistic	*p*	*partial* η^2
Genotype	F(1,39)=0.010	0.922	0.000
Sex	F(1,39)=1.825	0.185	0.045
Genotype × Sex	F(1,39)=0.419	0.521	0.011

Immobility

	F statistic	*p*	*partial* η^2
Genotype	F(1,39)=0.801	0.376	0.020
Sex	F(1,39)=0.065	0.801	0.002
Genotype × Sex	F(1,39)=0.570	0.455	0.014

Climbing

	F statistic	*p*	*partial* η^2
Genotype	F(1,39)=0.403	0.529	0.010
Sex	F(1,39)=0.626	0.434	0.016
Genotype × Sex	F(1,39)=0.134	0.716	0.003

Latency to 1st Immobility

	F statistic	*p*	*partial* η^2
Genotype	F(1,39)=0.356	0.554	0.009
Sex	F(1,39)=1.051	0.312	0.026
Genotype × Sex	F(1,39)=0.003	0.957	0.000

Supplemental Table S11. Two-way ANOVAs for behaviors during swim stress in Phase 1 Context mice.

Phase 1 Context

Swimming

	F statistic	*p*	*partial η^2*
Genotype	F(1,36)=0.013	0.911	0.000
Sex	F(1,36)=4.250	0.047	0.106
**Genotype × Sex**	**F(1,36)=5.572**	**0.024**	**0.134**

Immobility

	F statistic	*p*	*partial η^2*
Genotype	F(1,36)=0.283	0.598	0.008
Sex	F(1,36)=0.181	0.673	0.005
Genotype × Sex	F(1,36)=1.360	0.251	0.036

Climbing

	F statistic	*p*	*partial η^2*
Genotype	F(1,36)=0.765	0.388	0.021
Sex	F(1,36)=1.538	0.223	0.041
Genotype × Sex	F(1,36)=0.189	0.667	0.005

Latency to 1st Immobility

	F statistic	*p*	*partial η^2*
Genotype	F(1,36)=0.151	0.700	0.004
Sex	F(1,36)=0.259	0.614	0.007
Genotype × Sex	F(1,36)=2.022	0.164	0.053

Supplemental Table S12. Two-way ANOVAs for behaviors during swim stress in Phase 2 Cued mice.

Phase 2 Cued

Swimming

	F statistic	***p***	***partial η²***
Genotype	F(1,42)=1.062	0.309	0.025
Sex	F(1,42)=0.048	0.828	0.001
Genotype × Sex	F(1,42)=0.271	0.605	0.006

Immobility

	F statistic	***p***	***partial η²***
Genotype	*F(1,42)=3.221*	*0.080*	*0.071*
Sex	F(1,42)=0.065	0.800	0.002
Genotype × Sex	F(1,42)=1.161	0.287	0.027

Climbing

	F statistic	***p***	***partial η²***
Genotype	*F(1,42)=3.024*	*0.089*	*0.067*
Sex	F(1,42)=0.867	0.357	0.020
Genotype × Sex	F(1,42)=1.483	0.230	0.034

Latency to 1st Immobility

	F statistic	***p***	***partial η²***
**Genotype**	**F(1,42)=4.679**	**0.036**	**0.100**
Sex	F(1,42)=2.722	0.106	0.061
Genotype × Sex	F(1,42)=0.030	0.863	0.001

Supplemental Table S13. Two-way ANOVAs for behaviors during swim stress in Phase 2 Context mice.

Phase 2 Context

Swimming

	F statistic	*p*	*partial η^2*
Genotype	F(1,34)=0.694	0.411	0.020
**Sex**	**F(1,34)=4.996**	**0.032**	**0.128**
Genotype × Sex	F(1,34)=0.003	0.954	0.000

Immobility

	F statistic	*p*	*partial η^2*
Genotype	F(1,34)=1.512	0.227	0.043
**Sex**	**F(1,34)=10.09**	**0.003**	**0.229**
Genotype × Sex	F(1,34)=0.013	0.909	0.000

Climbing

	F statistic	*p*	*partial η^2*
Genotype	F(1,34)=0.930	0.342	0.027
**Sex**	**F(1,34)=5.611**	**0.024**	**0.142**
Genotype × Sex	F(1,34)=0.020	0.890	0.001

Latency to 1st Immobility

	F statistic	*p*	*partial η^2*
Genotype	F(1,34)=0.845	0.364	0.024
**Sex**	**F(1,34)=18.76**	**<0.001**	**0.356**
Genotype × Sex	F(1,34)=0.087	0.770	0.003

Supplemental Table S14. Two-way ANOVA of log-transformed corticosterone levels in mice 30 min following an acute swim stressor. Refer to Supplemental Figure S1.

**Log-transformed Corticosterone Levels**			
	F statistic	***p***	***partial η^2***
Genotype	F(2,62)=0.285	0.753	0.009
Sex	F(1,62)=0.368	0.546	0.006
Genotype × Sex	F(2,62)=0.706	0.497	0.022

Supplemental Table S15. Two-way ANOVAs of average of context fear expression during minutes 2-6. Refer to Supplemental Figures S2, S3.

**Phase 1 Cued**			
	F statistic	***p***	***partial η^2***
Genotype	F(1,88)=0.006	0.938	0.000
Sex	F(1,88)=1.056	0.307	0.012
Genotype × Sex	F(1,88)=0.196	0.659	0.002

**Phase 1 Context**			
	F statistic	***p***	***partial η^2***
**Genotype**	**F(1,77)=5.646**	**0.020**	**0.068**
Sex	F(1,77)=1.238	0.269	0.016
Genotype × Sex	F(1,77)=0.646	0.424	0.008

**Phase 2 Cued**			
	F statistic	***p***	***partial η^2***
Genotype	F(1,88)=0.006	0.938	0.000
Sex	F(1,88)=1.056	0.307	0.012
Genotype × Sex	F(1,88)=0.196	0.659	0.002

**Phase 2 Context**			
	F statistic	***p***	***partial η^2***
Genotype	F(1,88)=0.006	0.938	0.000
Sex	F(1,88)=1.056	0.307	0.012
Genotype × Sex	F(1,88)=0.196	0.659	0.002

Supplemental Table S16. Two-way ANOVAs of fecal boli during swim stress. Refer to Supplemental Figure S4.

**Phase 1 Cued**			
	F statistic	***p***	***partial η^2***
Genotype	F(1,45)=0.013	0.910	0.000
Sex	F(1,45)=0.015	0.902	0.000
Genotype × Sex	F(1,45)=0.604	0.441	0.013

**Phase 1 Context**			
	F statistic	***p***	***partial η^2***
Genotype	F(1,36)=0.032	0.858	0.001
Sex	F(1,36)=2.235	0.144	0.058
Genotype × Sex	F(1,36)=0.122	0.729	0.003

**Phase 2 Cued**			
	F statistic	***p***	***partial η^2***
Genotype	F(1,43)=1.661	0.204	0.037
**Sex**	**F(1,43)=6.354**	**0.016**	**0.129**
Genotype × Sex	F(1,43)=0.042	0.839	0.001

**Phase 2 Context**			
	F statistic	***p***	***partial η^2***
Genotype	F(1,34)=0.297	0.589	0.009
Sex	F(1,34)=1.126	0.296	0.032
Genotype × Sex	F(1,34)=1.126	0.296	0.032

Supplemental Table S17. Specific numbers of mice graphed in each Figure.

**Figure 2: Phase 1 Cued**	**Females**		**Males**	
	**Wildtype**	**Heterozygous**	**Wildtype**	**Heterozygous**
Training	20	19	25	33
Cued Expression Testing & Extinction Training	20	19	25	33
Extinction Retention Testing	20	19	25	32
Context Fear Expression	20	19	23	30
Cued Fear Renewal	20	19	22	31

**Figure 3: Phase 1 Context**	**Females**		**Males**	
	**Wildtype**	**Heterozygous**	**Wildtype**	**Heterozygous**
Training	20	18	20	23
Context Fear Expression	20	18	20	23

**Figures 4 & 5: Phase 2 Cued**	**Females**		**Males**	
Training	**Wildtype**	**Heterozygous**	**Wildtype**	**Heterozygous**
No Swim	11	11	9	11
Swim	12	11	9	11
Cued Expression Testing & Extinction Training				
No Swim	11	11	7	10
Swim	12	11	7	10
Extinction Retention Testing				
No Swim	10	9	9	11
Swim	10	10	10	12
Context Fear Expression				
No Swim	11	11	9	11
Swim	12	11	10	12
Cued Fear Renewal				
No Swim	10	11	7	10
Swim	11	11	10	12

**Figures 6 & 7: Phase 2 Context**	**Females**		**Males**	
Training	**Wildtype**	**Heterozygous**	**Wildtype**	**Heterozygous**
No Swim	9	10	9	9
Swim	10	9	9	10
Context Fear Expression				
No Swim	9	8	9	9
Swim	8	9	9	10

**Figure 8: Corticosterone**	**Females**		**Males**	
Phase 1 Cued	**Wildtype**	**Heterozygous**	**Wildtype**	**Heterozygous**
No Swim	10	8	12	15
Swim	10	10	15	16
Phase 1 Context				
No Swim	10	8	10	11
Swim	10	10	10	11

Figure 9: Corticosterone	Females		Males	
Phase 1 Cued	**Wildtype**	**Heterozygous**	**Wildtype**	**Heterozygous**
No Swim	11	11	10	11
Swim	12	11	12	12
Phase 1 Context				
No Swim	9	10	9	9
Swim	10	9	9	10

Figures 10 & 11: Swim behaviors	Females		Males	
	**Wildtype**	**Heterozygous**	**Wildtype**	**Heterozygous**
Phase 1 Cued	10	10	9	14
Phase 1 Context	10	10	9	11
Phase 2 Cued	12	11	12	11
Phase 2 Context	10	8	10	10

Supplemental Figure S1.

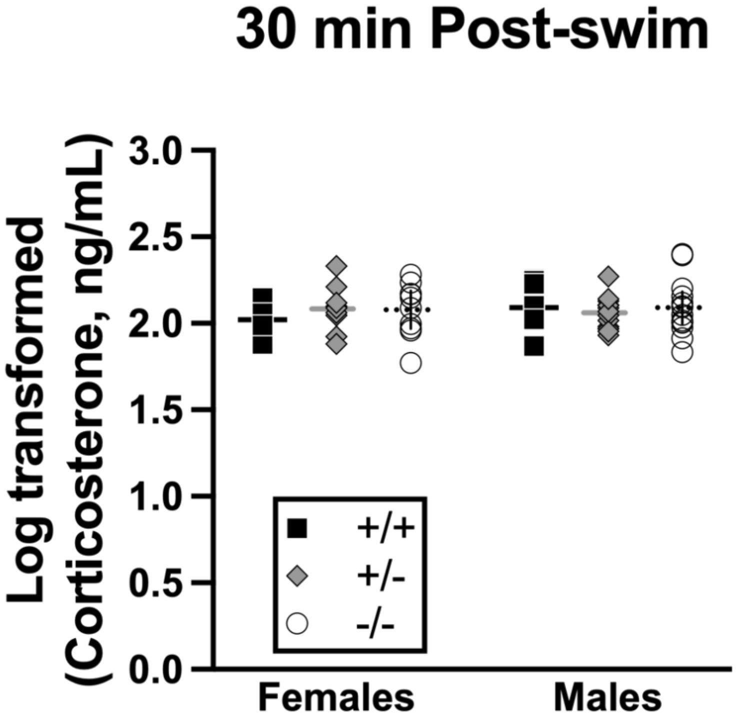

Supplemental Figure S1. ***Log-transformed corticosterone levels 30 min after an acute swim stress***.
Wildtypes are represented by black squares, heterozygotes by grey diamonds, and knockouts by clear circles. All mice underwent a six min swim stress, and blood was collected 30 min later to quantify serum corticosterone levels. Data are log-transformed cort levels [1–4]. Female wildtypes, n=10; female heterozygotes, n=9; female knockouts, n=10; male wildtypes, n=12; male heterozygotes, n=13, male knockouts, n=14. Data are graphed as mean (horizontal line) with 95% confidence interval (vertical line).

Supplemental Figure S2.

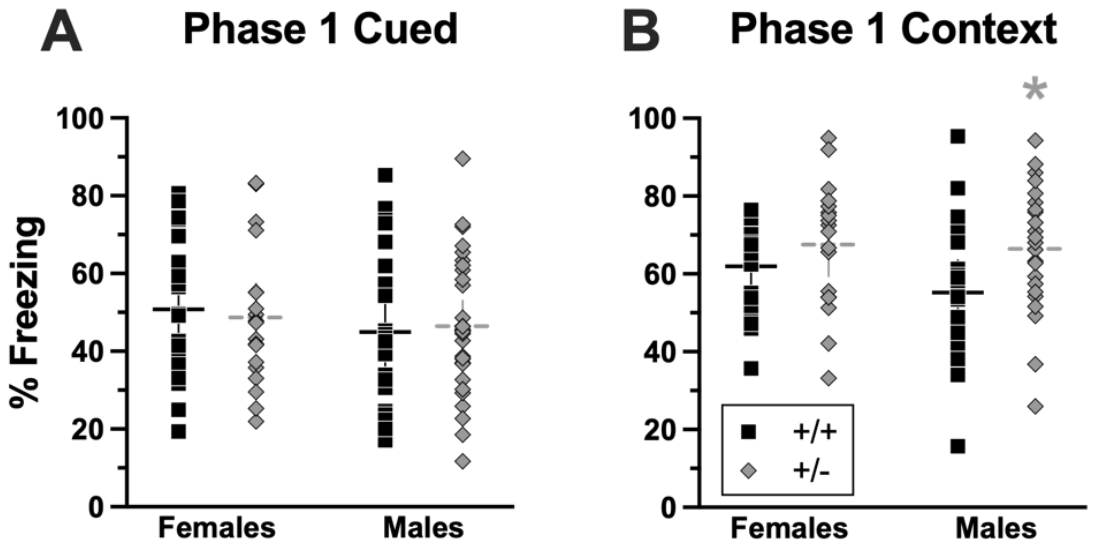

Supplemental Figure S2. ***Average context fear expression during minutes 2 through 6 of testing for Phase 1 mice***.
Wildtypes are represented by black squares, and heterozygotes are represented by grey diamonds. Context fear testing occurred on Day 5 for mice that underwent cued fear conditioning (A), whereas context fear testing occurred on Day 2 for mice that underwent context fear conditioning (B). Data are average percent time spent freezing during minutes two through six of the 10 min context fear testing period [2,5]. Phase 1 Cued: Female wildtypes, n=20; female heterozygotes, n=19; male wildtypes, n=23; male heterozygotes, n=30. Phase 1 Context: Female wildtypes, n=20; female heterozygotes, n=18; male wildtypes, n=20; male heterozygotes, n=23. Data are graphed as mean (horizontal line) with 95% confidence interval (vertical line). *p=0.0455 indicates difference between heterozygous and wildtype within same Phase 1 Context sex.

Supplemental Figure S3.

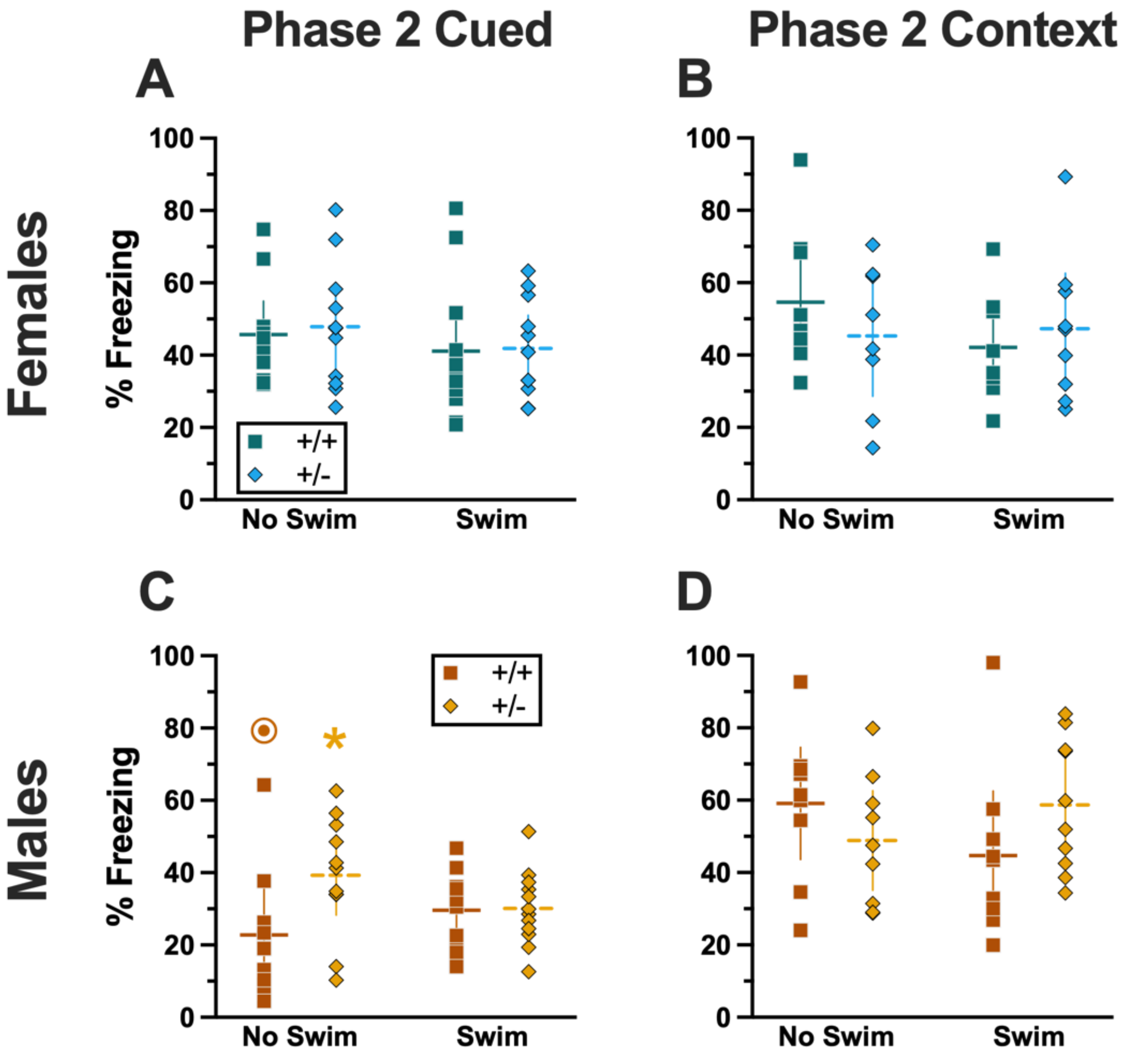

Supplemental Figure S3. ***Average context fear expression during minutes 2 through 6 of testing for Phase 2 mice***.
Female (A-B) wildtypes are represented by teal squares, and female heterozygotes are represented by blue diamonds. Male (C-D) wildtypes are represented by orange squares, and male heterozygotes are represented by yellow diamonds. Context fear testing occurred on Day 5 for mice that underwent cued fear conditioning (A,C), whereas context fear testing occurred on Day 2 for mice that underwent context fear conditioning (B,D). Data are average percent time spent freezing during minutes two through six of the 10 min context fear testing period [2,5]. Phase 2 Cued No Swim: Female wildtypes, n=11; female heterozygotes, n=11; male wildtypes, n=9; male heterozygotes, n=11. Phase 2 Cued Swim: Female wildtypes, n=12; female heterozygotes, n=11; male wildtypes, n=10; male heterozygotes, n=12. Phase 2 Context No Swim: Female wildtypes, n=9; female heterozygotes, n=8; male wildtypes, n=9; male heterozygotes, n=9. Phase 2 Context Swim: Female wildtypes, n=8; female heterozygotes, n=9; male wildtypes, n=9; male heterozygotes, n=10. Data are graphed as mean (horizontal line) with 95% confidence interval (vertical line). *p=0.019 indicates difference between

heterozygous and wildtype within same sex and swim condition. $^{\odot}p=0.001$ indicates difference between sexes within same genotype and swim condition.

Supplemental Figure S4.

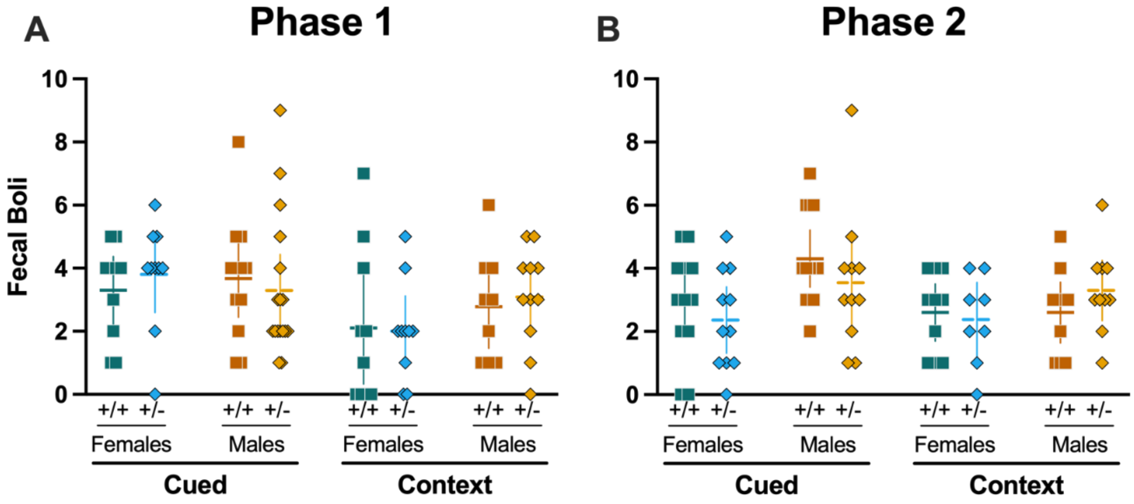

Supplemental Figure S4. ***Fecal boli from swim stressors across Phases***. Female wildtypes are represented by teal squares, and female heterozygotes are represented by blue diamonds. Male wildtypes are represented by orange squares, and male heterozygotes are represented by yellow diamonds. Mice were exposed to six minutes of swim stress after (Phase 1) or before (Phase 2) cued or context fear conditioning. Data are fecal boli produced during swim stress. Phase 1 Cued: Female wildtypes, n=10; female heterozygotes, n=10; male wildtypes, n=12; male heterozygotes, n=17. Phase 1 Context: Female wildtypes, n=10; female heterozygotes, n=10; male wildtypes, n=9; male heterozygotes, n=11. Phase 2 Cued: Female wildtypes, n=12; female heterozygotes, n=11; male wildtypes, n=13; male heterozygotes, n=11. Phase 2 Context: Female wildtypes, n=10; female heterozygotes, n=8; male wildtypes, n=10; male heterozygotes, n=10. Data are graphed as mean (horizontal line) with 95% confidence interval (vertical line).

Excluded fear behavior data

Phase 1 Cued	Reason
0608	All fear data excluded because cued fear expression never exceeds 25%
0609	Data are excluded for context fear testing & cued fear renewal because camera was laggy for 609, 610, and 616, thus freezing data are inaccurate
0610	Data are excluded for context fear testing & cued fear renewal because camera was laggy for 609, 610, and 616, thus freezing data are inaccurate
0616	Data are excluded for context fear testing & cued fear renewal because camera was laggy for 609, 610, and 616, thus freezing data are inaccurate
0664	First 5 tones of cued fear expression <25%, so all data excluded
0681	All fear data excluded because cued fear training never exceeds 25%
0768	First 5 tones of cued fear expression <25%, so all data excluded
0770	No freezing during context testing or cued fear renewal>25%
1057	No freezing during context testing >25%, context testing data excluded
1182	All fear data excluded because cued fear training never exceeds 25%
1168	Cued fear renewal data excluded because cued fear renewal never exceeds 25%
0828	First 5 tones of cued fear expression <25%, so all data excluded
1255	First 5 tones of cued fear expression <25%, so all data excluded
1264	First 5 tones of cued fear expression <25%, so all data excluded
1284	First 5 tones of cued fear expression <25%, so all data excluded

Phase 1 Context	Reason
0713	Shock did not work, was accidentally turned off; exclude all data because never trained
0771	Excluding data because mouse exhibited >75% freezing prior to first shock
0775	Excluding data because mouse exhibited >75% freezing prior to first shock
0776	No freezing during testing >25%, all data excluded
0832	No freezing during testing >25%, all data excluded
1081	Exceeded 75% freezing prior to first shock being administered; excluding all data
1070	No freezing during testing >25%, all data excluded
1080	No freezing during testing >25%, all data excluded

Phase 2 Cued	Reason
0782	All data excluded because first 5 tones cued fear expression on first testing day never exceed 25%
0792	Cued fear renewal data excluded because cued fear renewal never exceeds 25%
0796	Cued fear renewal data excluded because cued fear renewal never exceeds 25%
0827	Mice were not run under correct protocol for testing day 2, so excluding data from this testing day (were run under context testing/cued fear renewal protocol, though in context B)
0869	Context testing & cued fear renewal testing, door was open and mouse left the chamber at 532.67, data from 510 s onwards on this day excluded
0870	Cued fear renewal data excluded because cued fear renewal never exceeds 25%
0873	Mice were not run under correct protocol for testing day 2, so excluding data from this testing day (were run under context testing/cued fear renewal protocol, though in context B)
0879	Mice were not run under correct protocol for testing day 2, so excluding data from this testing day (were run under context testing/cued fear renewal protocol, though in context B)
0881	Videos did not record for cued expression testing & extinction training (7 May 2022) for these animals, so must exclude from analyses
0885	Mice were not run under correct protocol for testing day 2, so excluding data from this testing day (were run under context testing/cued fear renewal protocol, though in context B)
0886	Mice were not run under correct protocol for testing day 2, so excluding data from this testing day (were run under context testing/cued fear renewal protocol, though in context B)
0887	Mice were not run under correct protocol for testing day 2, so excluding data from this testing day (were run under context testing/cued fear renewal protocol, though in context B)
0908	Videos did not record for cued expression testing & extinction training (7 May 2022) for these animals, so must exclude from analyses
0909	Videos did not record for cued expression testing & extinction training (7 May 2022) for these animals, so must exclude from analyses
0910	Videos did not record for cued expression testing & extinction training (7 May 2022) for these animals, so must exclude from analyses
0916	Videos did not record for cued expression testing & extinction training (7 May 2022) for these animals, so must exclude from analyses
0917	Videos did not record for cued expression testing & extinction training (7 May 2022) for these animals, so must exclude from analyses; Cued fear renewal data excluded because cued fear renewal never exceeds 25%
0918	Videos did not record for cued expression testing & extinction training (7 May 2022) for these animals, so must exclude from analyses
0919	Videos did not record for cued expression testing & extinction training (7 May 2022) for these animals, so must exclude from analyses

0923	All data excluded because cued fear expression on first testing day never exceeds 25%
0924	Training video did not save/record; All data excluded because cued fear expression on first testing day never exceeds 25%
0925	Training video did not save/record
0929	Training video did not save/record; All data excluded because cued fear expression on first testing day never exceeds 25%
0944	Training video did not save/record
0945	All data excluded because cued fear expression on first testing day never exceeds 25%
0946	All data excluded because cued fear expression on first testing day never exceeds 25%
0953	Exclude all fear data. These animals were swam in both cohorts 19 and 20. Only Swim scoring of cohort 19 will be used.
0967	Exclude all fear data. These animals were swam in both cohorts 19 and 20. Only Swim scoring of cohort 19 will be used.
0981	All data excluded because cued fear expression to first 5 tones on first testing day never exceeds 25%
1192	All data excluded because first 5 tones cued fear expression on first testing day never exceed 25%
1210	All fear data excluded because cued fear training never exceeds 25%
1218	Cued fear renewal data excluded because cued fear renewal never exceeds 25%
1212	All data excluded because cued fear expression to first 5 tones on first testing day never exceeds 25%
1223	All fear data excluded because cued fear training never exceeds 25%

**Phase 2 Context**	**Reason**
1083	All data excluded because context fear expression never exceeds 25%
1092	Testing data didn't record because hard drive was not cleared on schedule
1098	Testing data didn't record because hard drive was not cleared on schedule
1079	Testing data didn't record because hard drive was not cleared on schedule
1106	Testing data didn't record because hard drive was not cleared on schedule
1141	Exceeded 75% freezing prior to first shock being administered; excluding all data

Excluded swim data

Male · Phase 1 Context · Wildtype<br>Climbing Behavior · (All Swim Data Excluded for 0836)			
**Animal ID**	**Data**	**Outlier Determination**	
0700	44.1		
0723	0.0	22.0	*Avg*
0726	16.6	16.7909946	*SD*
0783	28.5	105.92164	*Avg + 5 SD*
0819	4.6		
0820	0.9		
0836	121.5	>5 SD beyond mean	
0857	36.4		
1120	34.0		
1146	32.6		

Male · Phase 2 Cued · Wildtype<br>Swimming Behavior · (All Swim Data Excluded for 0944)			
**Animal ID**	**Data**	**Outlier Determination**	
0881	119.9		
0909	177.1		
0919	138.1		
0944	356.9	>8 SD beyond mean	
0946	172.1		
0952	118.3		
0967	159.4		
1009	151.4		
1034	132.4	135.1	*Avg*
1036	104.2	25.7674959	*SD*
1038	130.0	341.269134	*Avg + 8 SD*
1045	Recording failed		
1212	124.8		
1223	93.9		

Male · Phase 2 Cued · Heterozygous<br>Climbing Behavior · (All Swim Data Excluded for 0869)		
**Animal ID**	**Data**	**Outlier Determination**
0869	153.3	>5 SD beyond mean
0908	4.0	
0910	11.1	
0899	22.8	
0904	63.2	

0925	34.1		
0935	32.5		
0994	11.5		
1007	22.3	33.6	*Avg*
1016	31.6	23.2230254	*SD*
1086	67.2	149.706036	*Avg + 5 SD*
1125	69.2		

Female・Phase 2 Context・Heterozygous
Climbing Behavior・(All Swim Data Excluded for 0955)

Animal ID	Data	Outlier Determination	
0921	25.9		
0922	4.4	14.4	*Avg*
0937	0.5	15.7784437	*SD*
0943	44.5	140.65255	*Avg + 8 SD*
0949	1.4		
0955	144.0	>8 SD beyond mean	
1023	25.4		
1092	7.4		
1158	5.9		

References

1. Gilman, T.L.; George, C.M.; Andrade, M.A.; Mitchell, N.C.; Toney, G.M.; Daws, L.C. High Salt Intake Lowers Behavioral Inhibition. *Front Behav Neurosci* **2019**, *13*, 271, doi:10.3389/fnbeh.2019.00271.

2. Beaver, J.N.; Weber, B.L.; Ford, M.T.; Anello, A.E.; Ruffin, K.M.; Kassis, S.K.; Gilman, T.L. Generalization of Contextual Fear Is Sex-Specifically Affected by High Salt Intake. *PLOS ONE* **2023**, *18*, e0286221, doi:10.1371/journal.pone.0286221.

3. Uarquin, D.G.; Meyer, J.S.; Cardenas, F.P.; Rojas, M.J. Effect of Overcrowding on Hair Corticosterone Concentrations in Juvenile Male Wistar Rats. *J Am Assoc Laboratory Animal Sci Jaalas* **2016**, *55*, 749–755.

4. Teilmann, A.C.; Kalliokoski, O.; Sørensen, D.B.; Hau, J.; Abelson, K.S.P. Manual versus Automated Blood Sampling: Impact of Repeated Blood Sampling on Stress Parameters and Behavior in Male NMRI Mice. *Lab Anim* **2014**, *48*, 278–291, doi:10.1177/0023677214541438.

5. Lynch, J.F.; Winiecki, P.; Gilman, T.L.; Adkins, J.M.; Jasnow, A.M. Hippocampal GABAB(1a) Receptors Constrain Generalized Contextual Fear. *Neuropsychopharmacol* **2017**, *42*, 914–924, doi:10.1038/npp.2016.255.
